# Development and Psychometric Validation of the Athletes’ Perceived Body Pressures from Coaches Questionnaire (APBPCQ)

**DOI:** 10.3390/ijerph192416416

**Published:** 2022-12-07

**Authors:** Kaitlyn M. Eck, Carol Byrd-Bredbenner

**Affiliations:** 1 Department of Nutrition and Dietetics, Marywood University, Scranton, PA 18509, USA; 2 Department of Nutritional Sciences, Rutgers University, New Brunswick, NJ 08901, USA

**Keywords:** coaches, athletes, body concerns, perceived pressure, questionnaire, psychometrics

## Abstract

This study aimed to develop a questionnaire to assess male and female athletes’ perceived weight and shape pressure from coaches and establish its psychometric properties. Exploratory factor analysis (*N* = 412 in each female sample 1 and 2) provided evidence for 4 scales for female athletes and 3 scales for male athletes which were confirmed in confirmatory factor analysis (*N* = 260 in each male sample 1 and 2). For both females and males, KMO testing and Bartlett’s test of sphericity indicated that the sampling was adequate and survey items were appropriate for factor analysis. Additionally, all scales for both sexes had strong factor loadings (≥0.65), good Cronbach alpha coefficients (>0.70), and made contextual sense. The magnitude of difference results were indicative of a stable factor structure. Goodness-of-fit indicators were all in the expected direction. Good convergent validity was demonstrated. The questionnaire’s excellent psychometric properties and novelty make it a valuable tool for researchers and practitioners. This questionnaire has the potential to identify training needs in coaching staff, as well as to identify athletes who may benefit from support and guidance for effectively coping with pressure from coaches.

## 1. Introduction

Athletes, particularly those in aesthetic (e.g., cheerleading, diving, gymnastics, synchronized swimming, figure skating), leanness (e.g., cross country, track, swimming), and weight class (e.g., rowing, crew, wrestling) sports, as well as those in power sports (e.g., football, power lifting) are at increased risk for developing disordered eating behaviors [1,2]. Unfortunately, these behaviors can escalate into eating disorders, such as anorexia nervosa and bulimia nervosa, and result in severe health consequences impacting the cardiovascular, endocrine, gastrointestinal, and neurological systems [3,4]. Disordered eating behaviors also can negatively impact performance, impair recovery, and increase the risk of injury in athletes.

Numerous unique factors contribute to the development of disordered eating in athletes. These include the revealing nature of required uniforms, strict weight class cut offs, compulsory weigh-ins, subjective judgement based on aesthetics, perceptions held by athletes and coaches of performance advantages conferred by a particular body shape, weight, and/or composition, and remarks from influential individuals [5,6,7,8,9]. Comments from coaches, in particular, can have a considerable influence on athletes’ behaviors, including eating choices [10]. Negative words about an athlete’s body weight or shape made by coaches are associated with poorer body image and disordered eating behaviors in athletes [11,12]. Further, perceived pressure from coaches to achieve and/or maintain a particular body shape or weight positively correlates with symptoms of eating disorders in female athletes competing in aesthetic sports [13]. Similarly, male athletes whose coaches mandate weigh-ins engaged in more dietary restriction and have a greater preoccupation with body weight and composition [14].

Interviews of top collegiate female athletes from a variety of sports revealed that coaches make verbal observations about athletes’ body weight and/or shape for a variety of reasons [15]. The athletes perceived some remarks to be useful or necessary, such as commenting on an athlete’s body in general terms with the goal of “keeping bodies strong as opposed to conforming to body ideals” [15]. Other comments, however, were perceived as potentially harmful, including criticism and comparisons to other athletes [15].

Although pressure from coaches is associated with eating disorder risk in both males and females, the exact type of pressure differs by sex largely due to differences in body ideals for males and females [16]. Male college athletes report that the ideal male body is tall and muscular; ideally male athletes have a muscular upper body with a small waist [17]. Female athletes are encouraged to be thin, lean, and toned [18,19]. These differences in body image ideals suggest that the pressure placed on athletes likely differs by gender.

While pressure from coaches is a potential factor influencing eating behaviors and eating disorder risk in athletes, validated questionnaires assessing athletes’ experiences with pressure from coaches are rare. The Weight Pressures in Sport questionnaires for Males (WPS-M) and Females (WPS-F) were developed to assess sport-specific pressures experienced by athletes regarding weight, body shape and size, and appearance [6,16,20]. The questionnaire for males includes three scales (i.e., coach/teammate pressures about weight, importance of body weight and appearance, and pressures about weight and body due to the fit of sport uniforms) [16]. The questionnaire for females has two scales (i.e., coach and sport pressures about weight and pressures regarding appearance and performance) [20]. Both questionnaires contain items that assess pressure from coaches, however because these scales are intended to assess general sport-specific pressure, the coach-specific items are grouped into scales containing items assessing other types of pressure thereby negating the possibility of quantifying pressure specifically coming from coaches. Further, these scales were developed and validated for use in top elite collegiate athletes in the United States (i.e., termed “Division 1 athletes”), thus limiting their generalizability to other athletes.

There is an array of sport-specific pressures that may affect eating behaviors and eating disorder risk that are experienced by athletes. For example, pressures from revealing uniforms may be experienced by athletes in some in sports like swimming and gymnastics, but are of less concern in other sports, such as soccer and basketball. Pressures associated with weigh-ins required in a few sports, like wrestling and rowing, are not a factor in most other sports. Pressure from coaches, however, is a factor that transcends all sports. Further, this is a factor that can be improved through education of coaches. Having the ability assess perceived pressure from coaches has the potential to identify coach training needs as well as identify athletes who may need interventions designed to effectively cope with pressure and reduce disordered eating behaviors and eating disorder (e.g., anorexia nervosa) risk. Thus, the purpose of this study was to develop a questionnaire to measure male and female athletes’ perceived body weight and shape pressure from coaches and establish its psychometric properties.

## 2. Methods

The study was approved by the Institutional Review Board at the authors’ university. All participants gave informed consent.

### 2.1. Sample

College students at a large university in the northeastern United States were recruited to participate in an online survey that took approximately 30 min to complete. Participants were recruited via verbal and electronic announcements in general education courses (i.e., introductory nutrition, chemistry, biology, writing, math) and email announcements sent via official student listservs. The announcements recruited students to complete an online survey about student health thoughts and behaviors. Students were offered the opportunity to win 1 of 10 $25.00US gift cards in exchange for participation.

To be eligible for this component of this multifaceted study, participants had to be between the ages of 18 and 25 years, undergraduate students, and have attended high school in the United States to control for differences in educational systems. To ensure participants had experiences with coaches, they also had to have participated in a sport in high school and/or college. These athletes could be a current or former member of a school team, recreational league, club, or intramural team.

### 2.2. Instrument

The parts of the online survey pertinent to this study were demographic characteristics, height and weight, items assessing athletes’ perceived pressures from coaches related to body weight and shape, weight and shape concern scale items from the Eating Disorders Examination-Questionnaire (EDE-Q) [21], and the Achievement Orientation Questionnaire [22]. Demographic characteristics collected included biological sex, age, and race/ethnicity. Height and weight were used to calculate BMI using the standard formula.

The Athletes’ Perceived Body Pressures from Coaches Questionnaire (APBPCQ) items were created de novo based on interviews conducted with athletes and sports dietitians [23], expert input, the WPS-M and WPS-F questionnaires for males and females [6,16,20], and a review of published scales assessing pressure from parents, teachers, and other authority figures [24,25]. A bank of 50 items distributed across 4 conceptual areas (i.e., coaches notice athletes’ body weight/shape, coaches encourage athletes’ body weight/shape changes, coaches emphasize athletes’ body weight/shape links to performance, and coaches state expectations for athletes’ body shape/weight) were developed to measure the frequency (1 = never to 6 = always) athletes encountered each of these types of pressure. This Likert type answer format was selected because it provides a sufficient degree of accuracy and discrimination ability, and it is a familiar format thereby helping to reduce participant burden in completing the questionnaire and improve accuracy of responses [26]. These items were reviewed by a panel of three experts in nutrition, physical activity, athletics, coaching, and psychometric analysis. The panel of experts assessed the items for clarity, pertinence, and comprehensiveness, and reviewed the items for content validity. The initial review resulted in the elimination of 2 items identified as repetitive or not pertinent and suggestions for the addition of 6 items to improve comprehensiveness. Remaining items were refined to improve clarity. The resulting 54 items were again reviewed by the expert panel to confirm content validity. The items from the conceptual areas were interspersed in the survey in a random sequence to avoid a response set.

To establish convergent validity, the APBPCQ scales identified via factor analysis were scored by averaging the scores of the items in each factor. Then, the pressures scales were compared to suitable variables available in the data set (i.e., EDE-Q Shape Concerns and Weight Concerns scale items [21] and the Ego-Orientation and Goal-Orientation scales from the Achievement Orientation Questionnaire [22]). These variables were selected because research indicates that an emphasis on athletes’ body weight or shape by coaches is associated with poorer body image and concerns in athletes [11,12]. Other studies report that athletes in coaching environments that promote ego-orientation motivation (e.g., “I am clearly superior”) feel greater pressure to perform optimally, and are, therefore, more likely to frequently engage in behaviors such as dieting to control weight than those in goal-oriented environments (e.g., “I perform to the best of my ability”) [1]. Thus, it was hypothesized that those perceiving greater pressure from coaches would have higher shape and weight concerns, higher ego-orientation, and lower goal-orientation than those perceiving less pressure. The EDE-Q Shape and Weight Concerns scale items were scored on a 7-point frequency scale (1 = no days, 2 = 1–5 days, 3 = 6–12 days, 4 = 13–15 days, 5 = 16–22 days, 6 = 23–27 days, 7 = everyday), with higher scores indicating greater frequency of being concerned with shape and weight. The Ego-Orientation and Goal-Orientation scales contained 4- and 6-items, respectively, all of which were answered using a 5-point agreement scale ranging from strongly disagree to strongly agree [22]. Scale item scores were averaged to create scale scores; higher scores indicate a higher ego-orientation or goal-orientation, respectively [22].

### 2.3. Data Analysis

SPSS and AMOS version 28.0 (SPPS Inc., Chicago, IL, USA) were used to analyze study data. All analyses of the pressures from coach items were conducted separately for males and females because the type of pressure experienced by male and female athletes differs due to differences in body ideals by sex [16]. Each sex-specific data set was randomly split into two data sets, yielding Female Samples 1 and 2 and Male Samples 1 and 2. *T*-tests were performed to compare descriptive variables of the two female samples and of the two male samples to ensure groups were comparable. Kaiser-Meyer-Olkin (KMO) testing was conducted separately for each sex-specific random half to determine sampling adequacy for factor analysis, scores closer to 1 indicate greater adequacy of sampling [27]. Bartlett’s test of sphericity was conducted for each sex-specific random half to verify that the items were related and appropriate for factor analysis procedures, significance for this test was set at *p* < 0.01 [27,28].

Female Sample 1 and Male Sample 1 each were used separately for the exploratory factor analysis. Similarly, Female Sample 2 and Male Sample 2 were each used independently for the confirmatory factor analysis. Iterative exploratory principal components analysis with orthogonal (varimax) rotations were performed separately for Female Sample 1 and Male Sample 1 [29]. Exploratory factor analysis was conducted to establish factor structure and eliminate items with weak (<0.60) or cross (>0.40) factor loading coefficients one at a time. After each item deletion, the Cronbach alpha coefficient was determined to confirm internal consistency was acceptable (>0.70) [30] and remaining items made contextual sense. The systematic item elimination continued until factor loadings for all items was high (≥0.65), Cronbach alpha was acceptable, items made contextual sense, and at least 2 items remained in each factor [29]. At that point, confirmatory factor analysis was conducted independently with Male Sample 2 and Female Sample 2. In the confirmatory analysis the number of factors was stipulated to confirm the factor structure found in the exploratory analysis [31,32]. The magnitude of difference in each item’s factor loading from the confirmatory principal components analysis was compared across the sex-specific data sets. Magnitude of difference was calculated by squaring the difference between the factor loading for corresponding items in Female (or Male) Sample 1 and 2. Magnitude differences of ≤0.05 were considered to be small and indicative of a stable factor structure [33].

Using SPSS AMOS, Goodness-of-fit indicators (e.g., comparative fit index [CFI], goodness-of-fit index [GFI], Tucker–Lewis Index [TLI], root mean square error of approximation [RMSEA] and related 90% confidence interval [CI], and standard root mean-square residual [SRMR]) were conducted to verify the factor structure for the combined Female Samples 1 and 2 and the combined Male Samples 1 and 2. Goodness-of-fit indicators can range from 0 to 1 [29,34,35], with higher scores indicating better fit for all indicators except for RMSEA and SRMR for which values closer to 0 indicate better fit [35]. Chi-square and degrees of freedom were calculated; however, they were not considered as indicators of absolute fit due to the large sample sizes [35,36].

To determine convergent validity, independent 2-tailed *t*-tests were conducted to compare the scores on each APBPCQ scale resulting from the factor analyses described above to the EDE-Q Shape Concerns and Weight Concerns [21] and the Ego-Orientation and Goal-Orientation scores [22]. Cohen’s D was calculated to indicate effect sizes of significantly different results from *t*-test comparisons, with effect sizes of 0.2, 0.5, and 0.8 indicating small, medium, and larges affects, respectively [37]. The EDE-Q scores were dichotomized using a median split into low vs. high concerns. The Ego-Orientation and Goal-Orientation scores also were dichotomized using a median split into low vs. high ego-orientation and low vs. high goal-orientation.

## 3. Results

### 3.1. Participants

Of the 2035 survey participants, 1344 met eligibility criteria for this analysis. The analytic sample was comprised of 824 (61%) females and 520 (39%) males. As shown in Table 1, Female Samples 1 and 2 were about 20 years old, slightly more than half were White, BMI was in the healthy range, and three-quarters were high school athletes and a quarter were athletes in college. The two samples were comparable on all of these characteristics.

For male participants, both samples were about 20 years old, about two-thirds were White, and BMI was near the top of the range considered to be a healthy weight (i.e., 18 to 25) [38]. Six in 10 were high school athletes and nearly 4 in 10 were college athletes. No significant differences occurred between the two male samples.

### 3.2. Factor Analysis

KMO testing for both Female Samples 1 and 2 (i.e., 0.823, 0.777) and Male Samples 1 and 2 (i.e., 0.745, 0.737) revealed all were near 1 and thus, met the criteria for sampling adequacy [27]. Bartlett’s test of sphericity values were significant (*p* < 0.001) for both Female Samples 1 and 2 (i.e., χ^2^ = 4671.573, χ^2^ = 3875.094) and Male Samples 1 and 2 (i.e., χ^2^ = 1660.658, χ^2^ = 1466.751) indicating that the survey items were related and appropriate for factor analysis [27,28].

For Female Sample 1, iterative exploratory principal components analysis using the a priori elimination criteria yielded a reduction from 54 items to 13 items. This analysis generated 4 eigenvalues >1 thereby indicating a 4-factor solution. Confirmatory principal components analysis utilizing Female Sample 2 confirmed a 4-factor solution and confirmed the factor structure established by Female Sample 1 (Table 2). Cronbach alpha coefficients were >0.70, indicating that the items in all 4 factors, or APBPCQ scales, had acceptable internal consistency; expert review confirmed items within each APBPCQ scale made contextual sense [29]. Magnitude of difference for item factor loading between Female Sample 1 and Sample 2 ranged from 0.000 to 0.006, which were below the threshold of ≤0.05 indicating stable factor structure [31]. As seen in Table 3, goodness-of-fit values indicates all values are in the expected direction (i.e., CFI, GFI, TLI are close to 1 and SRMR and RMSEA are close to 0) [35].

For Male Sample 1, exploratory principal components analysis of all 52 items (male participants did not respond to 2 items of the original 54 items which were about menstruation) indicated a 3-factor solution. Confirmatory principal components analysis utilizing Male Sample 2 also indicated a 3-factor solution, confirming the factor structure established by Male Sample 1 (Table 4). Cronbach alpha coefficient were all acceptable (>0.70) indicating that APBPCQ scales had acceptable internal consistency and items included within each scale made contextual sense [29]. Magnitude of differences for item factor loadings between Male Sample 1 and Male Sample 2 ranged from 0.000 to 0.007, below the threshold of ≤0.05 indicating stable factor structure [31]. Goodness-of-fit indicators were similar to those reported for females (Table 5). That is, CFI, GFI, and TLI were closer to 1 while SRM, and RMSEA values were closer to 0 [35].

### 3.3. Convergent Validity

Independent *t*-tests conducted to establish convergent validity indicated that females in the high median split group for both the EDE-Q weight concerns and shape concerns scored significantly than those in the lower median split on the APBPCQ Scales 1 and 2 with a large effect size; no differences were noted for scales 3 and 4 (Table 6). As shown in Table 7, the results for males parallel those of females in that those with high weight and shape concerns scored significantly higher than comparators on Scales 1 and 2, but not Scale 3.

A comparison of high and low median split female groups for Ego-Orientation did not differ for any of the APBPCQ scales. However, this same comparison for males revealed significant differences for scale 1 with scale 3 approaching significance. A comparison of high and low median split female groups for the Goal-Orientation Scale revealed that females with higher goal-orientation scored significantly lower on APBPCQ Scales 1, 3, and 4 than those with low goal-orientation. This same comparison for males revealed no differences between the high and low goal-orientation groups for any APBPCQ scales.

## 4. Discussion

This study aimed to develop a questionnaire to assess male and female athletes’ perceived weight and shape pressure from coaches and establish its psychometric properties. This research indicated evidence for 4 APBPCQ scales for female athletes and 3 scales for male athletes. All scales for both sexes had strong factor loadings thereby indicating each was measuring a unique construct related to pressure from coaches. All scales had Cronbach coefficients indicative of good to excellent internal consistency [30]. Expert review confirmed content validity of all scales. Further, good convergent validity was demonstrated.

An array of goodness-of-fit indicators are reported in this paper to allow for comparisons across measures and guard against selective use of fit measures [39]. All goodness-of-fit indicators were all in the expected direction, however interpretation of these values is complex. Some advocate for strict application of cutoffs for labeling outcomes as a “good” or “bad” fit [40,41,42,43,44] (e.g., over the years, various values ranging upward from ≥0.9 have been put forth as indicative of ‘good’ fit for CIF, GFI, and TLI and ranging downward from <0.8 for RMSEA and SRMR), see for example [40,45,46]). Using these cutoffs, CFI and SRMR indicate “good fit” whereas others fall short by 0.01 to 0.05. However, other researchers feel cutoff values are arbitrary, lacking in empirical support, are not “golden rules” or tests of significance, are not universally appropriate, and/or should be abandoned [47,48,49,50,51,52]. The cutoff criteria used by some have been referred to as “merely guidelines” [53] or reference points [52]. Still other experts advise that “standard rules of thumb cannot be used without first considering the qualities of the data” [50], such as sample size, number of factors, strength of factor loadings, and number of variables—and because of the effects of these factors cutoff criteria should be used cautiously [52]. For example, χ^2^ (df) is almost always significant for samples the size of those in this study [35,36]. Similarly, CFI, TLI, and RMSEA are functions of χ^2^ and, thus, are affected by sample size [52]. SRMR and GFI also are sensitive to sample size [52], with some stating this and other drawbacks indicate GFI should not be used at all, especially with large sample sizes [41,54]. Given the effect of sample size, some scholars have proposed adusting cutoffs to account for sample size, yet the methodology for achieving this remains to be developed [52]. RMSEA is sensitive to total number of items and item quality, generating lower values with 30 or more items having lower factor loadings (i.e., 0.4) [55]. Thus, some suggest RMSEA should not be computed with data such as those in this study—that is, a small number (e.g., 10) of high quality (e.g., factor loadings of ~0.80) items [55,56]. Additionally, if RMSEA is computed, caution is needed when interpreting results [55]. Similarly, researchers are urged to pay close attention when interpreting CFI and TLI when applied to a small number of high-quality items [55].

When it comes to applying cutoffs, it is important to also recognize that an acceptable fit is does not automatically ensure a model is plausible [57]. Indeed, Stone advises that, in addition to empirical methods to determine a model’s fit, researchers also apply theory and logic [39]. This is vital in that “a model that is weakly justified theoretically but fits the data well (i.e., solely empirically driven) may not be a model of the hypothesized phenomenon that is as valid as a model that does not fit the data as well but has stronger theoretical support” [39]. Although the scales in this study, for both males and females, may fall somewhat short of commonly used but not universally accepted thresholds for some goodness-of-fit indicators, they have strong contextual sense reflective of the hypothesized phenomena. Thus, the authors contend that the APBPCQ for both males and females demonstrate acceptable model fit. Future research should aim to further the work of the questionnaires developed in this study to add to the body of evidence regarding goodness-of-fit and their utility in assessing the constructs identified and developing intervention programs to lessen perceived pressures from coaches.

Items on the APBPCQ for females represent the 4 domains of athletes’ perceived pressure from coaches: coaches’ beliefs about athletes’ body weight and shape (Scale 1), coaches’ role related to athletes’ body weight and shape (Scale 2), coaches’ advice to athletes on body weight and shape (Scale 3), and coaches’ expectations of athletes’ body weight and shape (Scale 4). APBPCQ scale items for males represent 3 domains of pressure: coaches’ focus on athletes’ body weight, shape, and muscle mass (Scale 1); coaches’ role related to athletes’ body weight, shape, and muscle mass (Scale 2); and coaches’ advice to athletes on body weight and shape (Scale 3). A comparison of the domains for females and males reveals that the Scale 1 has a similar thrust for both sexes, but items for males tend to be more neutral in tone (i.e., coach focuses on…) whereas those for females are more polarized (i.e., “coach encourages” vs. “coach pressures” and “unrealistic”). Other studies have shown that adolescent girls are more susceptible to peer pressure than boys, and are more likely to internalize problems and are more susceptible to psychological health concerns (e.g., depression, eating disorders) [58,59]. These gender difference may partially explain the more neutral nature of the male items. Scales 2 and 3 for males and females are similar except the scales for males also resulted in items related to muscle mass loading strongly. This difference is not surprising as body image ideals differ between males and females with a greater emphasis placed on muscularity for males [17,18,19]. Interestingly, a fourth factor emerged for females—this scale focused on the reasonableness of coaches’ expectations related to athletes’ weight and shape. These results support the need for sex specific scales for assessing pressure from coaches.

It is important to note that the 2 items on the APBPCQ Scale 4 for females are phrased positively whereas analogous items are phrased negatively on Scale 1 for females (i.e., reasonable vs. unrealistic expectations). The differential factor loading of positive and negative items has been reported by others (e.g., [60,61]) with various conjectures as to why this occurred, such as low reading skills in children, or simply being an artifact of factor analysis [61,62]. Future research should aim to examine whether Scale 4 for females yields information that is unique from Scale 1 and, if not, perhaps these scales can be combined and/or one of them deleted.

The study findings indicate that the hypothesis that those perceiving greater pressure from coaches would have higher shape and weight concerns is true. That is, those who had high shape or weight concerns perceived significantly greater pressure from coaches regarding coaches’ beliefs and behaviors as well as their roles related to athletes’ bodies. The higher perceptions of pressure from coaches among those in the upper median split for weight and shape concerns that were observed in this study align with previous research which has shown that an emphasis on athletes’ body weight or shape by coaches is associated with poorer body image in athletes [11,12]. The hypothesis that those perceiving greater pressure from coaches would have higher ego-orientation tended to be true only for males whereas the hypothesis that those perceiving greater pressure would have lower goal-orientation tended to be true only for females. Previous research indicates that stronger ego-orientation is related to more dieting and weight-related peer pressure among female aesthetic sports performers (i.e., gymnasts and dancers) [1]. The lack of relationship between ego-orientation and perceived pressure from coaches observed for females may be because a greater array of types of sports, individual and team sports, and competition levels (e.g., recreational leagues, intermural teams, Division I college teams) were represented in this study and the focus was on coach, rather than peer, pressures. The finding that males, but not females, with high ego-orientation tended to perceive greater pressure from coaches may be due to differences between the sexes vis à vis ego-orientation in that males tend to be socialized to be competitive [63]. Additionally, evidence suggests that males tend to be ego-oriented in sports whereas females tend to be more task or goal oriented [64,65]. The research reported here also implies that goal-orientation may lower the pressure from coaches felt by female athletes, but not their male counterparts. Thus, coaches may help female athletes to manage the pressures of competition by promoting a mindset of aiming to perform to the best of their abilities.

The APBPCQ represents an important addition to the literature–to the authors’ knowledge this is the first study to develop and psychometrically analyze a questionnaire specifically assessing pressure from coaches. Additionally, this questionnaire was created and validated with athletes competing in a variety of sports at various levels of competition which expands the focus of previous assessments that have focused only on elite athletes [6,16,20]. The APBPCQ allows for the evaluation of perceived pressure from coaches and can be used to identify possible training needs of coaches. Further, this questionnaire may be useful in reducing disordered eating behaviors by allowing for the identification athletes who may benefit from interventions to help them effectively cope with perceived pressure. The large, racially diverse sample is an important strength of this study; however all participants were enrolled at one large, public university in the northeastern United States, which may limit generalizability.

## 5. Conclusions

The APBPCQ’s excellent psychometric properties and novelty make it a valuable tool for researchers and practitioners. The APBPCQ has the potential to identify training needs in coaching staff, as well as to identify athletes who may benefit from support and guidance for effectively coping with pressure from coaches. Training coaches to reduce body pressuring as well as providing support to help athletes better able to cope with perceived pressure from coaches may aid in the reduction of disordered eating behaviors and eating disorder risk. Future studies should seek to validate the APBPCQ scales in other demographic groups, such as younger and older athletes, professional athletes, and those living in other countries. Future research also should aim to assess the relationship between perceived body pressure from coaches and disordered eating behaviors and eating disorder risk in male and female athletes and determine the effectiveness of interventions to reduce pressure for coaches and improve coping with pressure in athletes.

## Figures and Tables

**Table 1:** Comparison of Characteristics of Female and Male Data Sets 1 and 2.

Sample	Age	White/Caucasian	BMI	Athlete Classification	
	Mean ± SD	*N* (%)		High School Athlete *N* (%)	College Athlete *N* (%)
**Females**					
Sample 1 (*n* = 412)	20.26 ± 1.25	231 (56)	23.44 ± 4.61	304 (74)	108 (26)
Sample 2 (*n* = 412)	20.39 ± 1.34	228 (55)	23.17 ± 4.23	304 (74)	108 (26)
*p*-value (*t*-test)	0.154	0.833	0.362	1.00	1.00
**Males**					
Sample 1 (*n* = 260)	20.40 ± 1.35	173 (67)	24.09 ± 3.74	158 (61)	102 (39)
Sample 2 (*n* = 260)	20.47 ± 1.36	156 (60)	24.52 ± 4.15	158 (61)	102 (39)
*p*-value (*t*-test)	0.285	0.122	0.110	1.00	1.00

**Table 2:** Exploratory and Confirmatory Factor Loadings and Magnitude of Differences for Females’ Responses to Athletes’ Perceived Body Pressures from Coaches Questionnaire.

Scale Name Item	Factor Loading and Cronbach Alphas		Magnitude of Differences
	Exploratory Sample 1 (*n* = 412)	Confirmatory Sample 2 (*n* = 412)	
**Scale 1: Perception of coaches’ beliefs and behaviors related to athletes’ body weight and shape**	α = 0.957	α = 0.936	
My coach encourages athletes to drop weight, even if they are preforming well.	0.912	0.917	0.000
My coach pressures athletes to maintain a low body weight.	0.902	0.842	0.004
My coach encourages athletes to drop weight, even if they are not overweight.	0.902	0.915	0.000
My coach has unrealistic expectations of athletes’ body shape.	0.899	0.865	0.001
My coach pressures athletes to change their body shape.	0.893	0.817	0.006
My coach has unrealistic expectations for athletes’ body weights.	0.869	0.852	0.000
**Scale 2: Perception of coaches’ role related to athletes’ body weight and shape**	α = 0.872	α = 0.841	
It is the responsibility of a coach to give athletes’ weight loss advice.	0.898	0.884	0.000
It is the job of a coach to set weight loss goals for athletes.	0.862	0.849	0.000
If I were a coach, I would make it a point to talk to athletes about their body weight.	0.860	0.859	0.000
**Scale 3: Perception that coaches should not advise athletes on body weight and shape**	α = 0.743	α = 0.742	
Setting body shape standards for athletes is not something coaches should do.	0.890	0.882	0.000
It is not the business of coaches to tell athletes how much to weigh.	0.878	0.888	0.000
**Scale 4: Perception of coaches’ expectations of athletes’ body weight and shape**	α = 0.948	α = 0.952	
My coach’s expectations for athletes’ body weight are reasonable.	0.962	0.970	0.000
My coach’s expectations for athletes’ body shape are reasonable.	0.960	0.968	0.000

**Table 3:** Goodness-of-fit Indices for Athletes’ Perceived Body Pressures from Coaches Questionnaire for Females.

Scheme	Female Sample (*n* = 824)
χ^2^ (df) ^a^	839.989 (59) *
CFI	0.908
GFI	0.88
TLI	0.878
RMSEA (90% CI)	0.127 (0.119 to 0.135)
SRMR	0.0393

* *p* < 0.001. ^a^ DF, degrees of freedom; CFI, comparative fit index; GFI, goodness-of-fit index; TLI, Tucker–Lewis Index; RMSEA, root mean square error of approximation; CI, confidence interval; SRMR, standardized root mean-square residual.

**Table 4:** Exploratory and Confirmatory Factor Loadings and Magnitude of Differences for Male Responses to Athletes’ Perceived Body Pressures from Coaches Questionnaire.

Scale Name Item	Factor Loading and Cronbach Alphas		Magnitude of Differences
	Exploratory Sample 1 (*n* = 260)	Confirmatory Sample 2 (*n* = 260)	
**Scale 1: Perception of coaches’ focus on athletes’ body weight, shape, and muscle mass**	α = 0.938	α = 0.941	
My coach focuses on team members’ muscle mass, even if they are performing well.	0.923	0.927	0.000
My coach focuses on team members’ body shape, even if they are performing well.	0.910	0.918	0.000
My coach focuses on team members’ body weight, even if they are performing well.	0.909	0.885	0.000
My coach focuses on team members’ muscle mass, especially if they are performing poorly.	0.891	0.922	0.000
**Scale 2: Perception of coaches’ role related to athletes’ body weight, body shape, and muscle mass**	α = 0.818	α = 0.700	
My coach should not try to play a role in determining my body shape goals.	0.848	0.762	0.000
My body weight really isn’t any business of my coach.	0.844	0.847	0.000
It is not the business of coaches to tell athletes how much to weigh.	0.800	0.715	0.007
Setting body shape standards for athletes is not something coaches should do.	0.713	0.663	0.003
**Scale 3: Perception that coaches should not advise athletes on body weight, body shape, and muscle mass**	α = 0.851	α = 0.769	
It is the responsibility of a coach to give athletes weight loss advice.	0.910	0.885	0.001
If I were a coach, I would make it a point to talk to athletes about their body weight.	0.899	0.884	0.000

**Table 5:** Goodness-of-fit Indices for the Athletes’ Perceived Body Pressures from Coaches Questionnaire for Males.

Statistic	Male Sample (*n* = 520)
χ^2^ (df) ^a^	304.038 (32) *
CFI	0.911
GFI	0.893
TLI	0.875
RMSEA (90% CI)	0.128 (0.115–0.141)
SRMR	0.0275

* *p* < 0.001. ^a^ DF, degrees of freedom; CFI, comparative fit index; GFI, goodness-of-fit index; TLI, Tucker–Lewis Index; RMSEA, root mean square error of approximation; CI, confidence interval; SRMR, standardized root mean-square residual.

**Table 6:** Comparison of Athletes’ Perceived Body Pressures from Coaches Questionnaire (APBPCQ) by Weight Concern, Shape Concern, and Achievement Orientation in Females (*n* = 824).

APBPCQ Scales	High Median Split	Low Median Split	*p*-Value	Cohen’s D
	**Weight Concern**			
	**(*n* = 412)**	**(*n* = 412)**		
Scale 1: Perception of coaches’ beliefs and behaviors related to athletes’ body weight and shape	1.51 ± 0.88	1.22 ± 0.51	<0.001	0.718
Scale 2: Perception of coaches’ role related to athletes’ body weight and shape	2.34 ± 0.95	2.05 ± 0.90	<0.001	0.924
Scale 3: Perception that coaches should not advise athletes on body weight and shape	2.56 ± 1.09	2.61 ± 1.18	0.487	
Scale 4: Perception of coaches’ expectations of athletes’ body weight and shape *	1.80 ± 1.73	1.85 ± 1.91	0.747	
	**Shape Concern**			
	**(*n* = 418)**	**(*n* = 406)**		
Scale 1: Perception of coaches’ beliefs and behaviors related to athletes’ body weight and shape	1.50 ± 0.86	1.23 ± 0.54	<0.001	0.720
Scale 2: Perception of coaches’ role related to athletes’ body weight and shape	2.34 ± 0.96	2.05 ± 0.89	<0.001	0.924
Scale 3: Perception that coaches should not advise athletes on body weight and shape	2.55 ± 1.08	2.63 ± 1.18	0.315	
Scale 4: Perception of coaches’ expectations of athletes’ body weight and shape *	1.75 ± 1.71	1.90 ± 1.92	0.238	
	**Ego-Orientation**			
	**(*n* = 683)**	**(*n* = 140)**		
Scale 1: Perception of coaches’ beliefs and behaviors related to athletes’ body weight and shape	1.40 ± 0.93	1.36 ± 0.68	0.601	
Scale 2: Perception of coaches’ role related to athletes’ body weight and shape	2.26 ± 1.08	2.18 ± 0.90	0.435	
Scale 3: Perception that coaches should not advise athletes on body weight and shape	2.51 ± 1.24	2.60 ± 1.11	0.440	
Scale 4: Perception of coaches’ expectations of athletes’ body weight and shape *	1.86 ± 1.92	1.82 ± 1.80	0.808	
	**Goal-Orientation**			
	**(*n* = 449)**	**(*n* = 375)**		
Scale 1: Perception of coaches’ beliefs and behaviors related to athletes’ body weight and shape	1.32 ± 0.69	1.42 ± 0.78	0.029	0.731
Scale 2: Perception of coaches’ role related to athletes’ body weight and shape	2.22 ± 0.95	2.16 ± 0.92	0.183	
Scale 3: Perception that coaches should not advise athletes on body weight and shape	2.52 ± 1.14	2.67 ± 1.11	0.024	1.130
Scale 4: Perception of coaches’ expectations of athletes’ body weight and shape *	1.98 ± 1.88	1.64 ± 1.72	0.004	1.812

* Reverse scored.

**Table 7:** Comparison of Athletes’ Perceived Body Pressures from Coaches Questionnaire by Weight Concern, Shape Concern, and Achievement Orientation in Males (*n* = 520).

APBPCQ Scales	High Median Split	Low Median Split	*p*-Value	Cohen’s D
	**Weight Concern**			
	**(*n* = 280)**	**(*n* = 240)**		
Scale 1: Perception of coaches’ focus on athletes’ body weight, shape, and muscle mass	2.17 ± 1.25	1.88 ± 1.16	0.008	1.210
Scale 2: Perception of coaches’ role related to athletes’ body weight, body shape, and muscle mass	3.12 ± 0.96	2.68 ± 1.02	<0.001	0.989
Scale 3: Perception that coaches should not advise athletes on body weight, body shape, and muscle mass	2.91 ± 0.46	2.95 ± 0.45	0.339	
	**Shape Concern**			
	**(*n* = 271)**	**(*n* = 249)**		
Scale 1: Perception of coaches’ focus on athletes’ body weight, shape, and muscle mass	2.16 ± 1.25	1.90 ± 1.17	0.015	1.211
Scale 2: Perception of coaches’ role related to athletes’ body weight, body shape, and muscle mass	3.11 ± 0.96	2.70 ± 1.02	<0.001	0.992
Scale 3: Perception that coaches should not advise athletes on body weight, body shape, and muscle mass	2.91 ± 0.48	2.94 ± 0.43	0.513	
	**Ego-Orientation**			
	**(*n* = 263)**	**(*n* = 257)**		
Scale 1: Perception of coaches’ focus on athletes’ body weight, shape, and muscle mass	2.21 ± 1.29	1.86 ± 1.11	<0.001	1.21
Scale 2: Perception of coaches’ role related to athletes’ body weight, body shape, and muscle mass	2.97 ± 1.02	2.86 ± 1.00	0.112	
Scale 3: Perception that coaches should not advise athletes on body weight, body shape, and muscle mass	2.96 ± 0.45	2.89 ± 0.47	0.052	0.458
	**Goal-Orientation**			
	**(*n* = 294)**	**(*n* = 226)**		
Scale 1: Perception of coaches’ focus on athletes’ body weight, shape, and muscle mass	2.09 ± 1.22	1.96 ± 1.21	0.244	
Scale 2: Perception of coaches’ role related to athletes’ body weight, body shape, and muscle mass	2.93 ± 1.09	2.90 ± 0.91	0.782	
Scale 3: Perception that coaches should not advise athletes on body weight, body shape, and muscle mass	2.92 ± 0.48	2.93 ± 0.42	0.712	

## Data Availability

No new data were created or analyzed in this study. Data sharing is not applicable to this article.
